# Remembrance of things perceived: Adding thalamocortical function to artificial neural networks

**DOI:** 10.3389/fnint.2023.1108271

**Published:** 2023-03-07

**Authors:** Gerald E. Loeb

**Affiliations:** Alfred E. Mann Department of Biomedical Engineering, University of Southern California, Los Angeles, CA, United States

**Keywords:** thalamus, memory, learning, illusion, attention, saliency

## Abstract

Recent research has illuminated the complexity and importance of the thalamocortical system but it has been difficult to identify what computational functions it performs. Meanwhile, deep-learning artificial neural networks (ANNs) based on bio-inspired models of purely cortical circuits have achieved surprising success solving sophisticated cognitive problems associated historically with human intelligence. Nevertheless, the limitations and shortcomings of artificial intelligence (AI) based on such ANNs are becoming increasingly clear. This review considers how the addition of thalamocortical connectivity and its putative functions related to cortical attention might address some of those shortcomings. Such bio-inspired models are now providing both testable theories of biological cognition and improved AI technology, much of which is happening outside the usual academic venues.

## The general challenge

If you examine the contents of the smart phone that most people now carry, you will generally find a few hundred or perhaps thousand snapshots that the owner chose to memorialize. What was so special about them? During the ~16 h a day while we are awake, we make and process about three gaze shifts (saccades) per second, for a total of >100,000 visual images per day. Similarly, people make about three exploratory movements per second with their hands regarding any object or surface that they might contact. The decisions behind and the experiences of these elective events are an essential part of our ability to function in complex and largely unstructured human environments, yet they are almost completely subconscious. Nevertheless, a few of these events rise to conscious awareness and may be remembered, at least for a while in our mind’s eye and perhaps forever on our smart phones. Marcel Proust created his seven-volume novel *Remembrance of Things Past* out of the involuntary mental snapshots that provided the book’s fictional narrator with a fragmentary representation of his life. By using “remembrance” instead of “memory”, Proust and this review focus on the act of remembering and the decision-making that precipitates this act, as opposed to the form or content of memory.

We now understand that biological remembering comes in many forms, only some of which lead to vivid snapshots for conscious recall. Instead, most remembering consists of shifts in how we perceive the world in the future. Such shifts also affect the content that we recall as snapshots. Thus decisions about when and how to remember which aspects of experiences have large consequences for how we perceive and react to future events. Such decision-making has received relatively little recent attention in the burgeoning field of artificial intelligence. The dominant current models are ANNs that leave faint traces of every input that they receive, according to rules that are designed to distill passive experience into recurring patterns that can be given discrete labels—declarative memory. Preprocessors to amplify aspects of sensory data that might reflect salient features may modulate such traces, but these processes are continuous and automatic, essentially lacking in the decision-making aspects of human attention. Current thinking about thalamocortical function provides a broader and more dynamic view of salience that is starting to be incorporated in some ANN research.

The process of attending to a stimulus is often marked by a large, widespread and easily recorded electrical signal called the P300 wave in the electroencephalogram (EEG). This artifact signifies that some complex process has occurred, but it provides little more useful information about that process than the clicking shutter sound emitted by a digital camera when the user takes a snapshot (Donchin, 1981). The P300 is the consequence of a synchronization of synaptic currents at the cortical end of a recurrent loop between cerebral cortex and thalamus (Yingling and Hosobuchi, 1984; Hsu et al., 2018). How this is triggered and what it accomplishes remains speculative. The absence of analogous events and processes in machines claiming to be artificially intelligent may underlie their increasingly manifest limitations. Some of the enhancements to ANNs that are currently being developed were inspired by or might adventitiously shed light on thalamocortical function.

Much has been written about the role of the thalamocortical system in the many oscillatory rhythms that can be recorded in the EEG (Izhikevich and Edelman, 2008) and the role of synchronized spike activity in perception (Singer, 2009). Most computational models of thalamus have focused on the generation of such temporal patterns (Suffczynski et al., 2001; Muller and Destexhe, 2012; Willis et al., 2015; Bhattacharya et al., 2021) and their modification by clinical deep brain stimulation of the basal ganglia (Rubin and Terman, 2004; So et al., 2012; Yu et al., 2020). This review focuses on high-level connectivity and functionality without considering whether such temporal patterning is an essential mechanism or an epiphenomenon of that functionality. Much has been written about the role of the thalamocortical system in what philosophers call “consciousness” (Edelman and Tononi, 2000; Crick and Koch, 2003; Melloni et al., 2007). This review focuses on the various mechanisms and levels of attending to sensory events without proposing whether any of these are necessary or sufficient to account for the subjective states of consciousness or awareness.

## A general strategy

Much of what was first learned about brain function came from “experiments of nature” in which neuroscientists studied the disabilities of subjects with lesions of specific parts of their nervous systems. Much of what neuroscientists now hope to learn about brain function comes from building computational models and examining their emergent behaviors. Perhaps we can learn from the disabilities of these deficient “experiments of technology.” Furthermore, normal humans exhibit behaviors such as illusions that an AI engineer might consider to be defects to be overcome by inventing a better machine A scientist seeking to discover the substrate for human intelligence could use such quirks to differentiate among models that otherwise have similar capabilities (Loeb, 2022).

As David Marr pointed out, the interpretation of any experimental data regarding brain function depends on a top level “theory of computation” for how a capability is divided into a series of steps that might be performed by different parts of a brain or a machine (Marr, 1982). Such a theory might be wrong, but at least starting with a theory affords an escape from circular arguments like “the cerebellum does whatever it is that you can’t do when you don’t have one.” Neurophysiologists have long hoped that theories of computation would lead to testable hypotheses that might invalidate at least some theories, but the diffuse and recurrent nature of neural circuitry often makes such tests inconclusive. This is embodied by the cynical universal finding of neural recording that “some go up, some go down and some stay the same.” The theory of computation underlying an engineered machine or computer model is fully known (albeit not always clearly articulated). Differences in performance between such model systems and humans should provide a test of theories of neural computation, assuming that the machine implements its theory of computation adequately at the deeper levels that Marr identified as algorithm and hardware.

In the 73 years since Donald Hebb’s classic proposal regarding *The Organization of Behavior* (Hebb, 1949), increasingly ambitious implementations of neuromorphic machines such as deep learning ANNs have achieved remarkable success at specific tasks that were once thought to be hallmarks of uniquely human intelligence, e.g., identifying objects in complex scenes, interpreting running speech, playing strategic games. At the same time, we have come to realize that such capabilities do not generalize well to the robust interactions with unstructured physical environments that can be demonstrated by a 5-year-old child (Loeb, 2022). The engineers who built the machines that now outperform humans in tasks like playing chess may resent this “moving of the goalposts” for AI, but the need to do so is another aspect of the lack of a theory of computation. Apparently, we do not even understand *what* needs to be computed to achieve humanlike intelligence, much less *how* it is computed.

## The biological substrate

At the least, a bio-inspired theory of computation for an artificially intelligent machine would be expected to exhibit various capabilities and limitations that are well-known in humans but which have often been absent, elusive or overlooked in AI models. This review examines the thesis that various of these phenomena arise in the recurrent loops of the thalamocortical circuits that are not represented in most ANN models of intelligence. The connectivity and function of those circuits has been the subject of much research and speculation over the past decade. Some of the histologically distinct nuclei of the thalamus have clear associations with specific sensory modalities (lateral geniculate conveys visual information from retina, medial geniculate conveys auditory signals, ventroposterior nuclei convey somatosensory signals), but current views reject the original description of thalamic circuits as “relays” whereby sensory information is simply conveyed to cerebral cortex for all processing (Sherman, 2007). In general, whenever neural signals pass through synapses from one neuron to another, there is the opportunity for substantial transformation. This is particularly likely when those signals pass through interneurons that are also receiving recurrent feedback from the ultimate destination of the transformed signals, as is the case in all parts of the thalamus.

**Figure 1:**
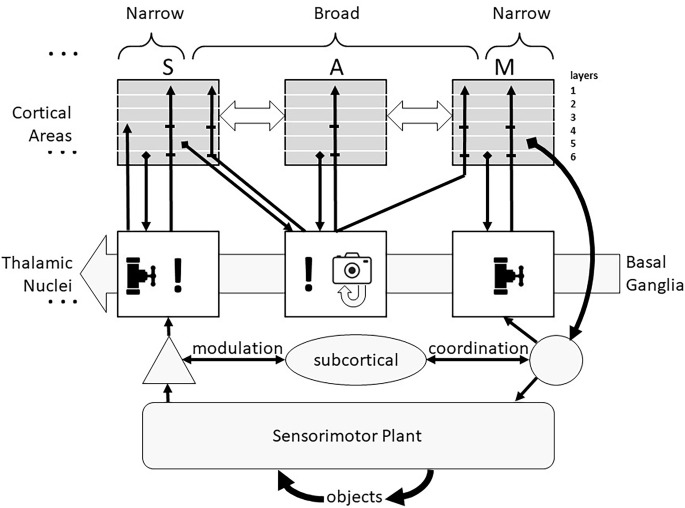
Corticothalamic recurrent circuits support similar computational functions in all cortical areas. The transmission of sensory information from all receptor modalities (except olfactory) to cortex is modulated in thalamus (valve symbol) and the excitability of the multiple primary sensory (S) cortical areas to that information is modulated by Narrow thalamocortical projections (!). If the sensory information is incongruent with any of the familiar expectations, corticothalamic activity leads to activation of Broad thalamocortical projections from non-sensory thalamic neurons that recruit cognitive processing by other sensory, associative (A) and motor (M) cortical areas, often culminating in the identification of an exploratory action to obtain additional sensory information about the unknown object. The thresholds for these thalamic recruitments of cortical functions (including overt exploratory actions) are set by the Basal Ganglia in consideration of a risk-benefit analysis. The Broad thalamocortical projections are also capable of Hebbian potentiation of their synapses onto currently active cortical neurons, providing a snapshot memory (camera icon) of the incongruent sensorimotor activity that can be replayed off-line by spontaneous activity in those thalamocortical neurons. The general locations of cortical inputs and outputs are shown by layers. The corticothalamic system adds sophisticated cognitive decision-making to a subcortical system (tectum, reticulospinal system, and cerebellum) that is itself capable of fast and accurate sensorimotor performance. Collateral discharge from this system passes through thalamic nuclei to motor cortical areas.

A more general and functional view of the thalamus (see Figure 1) divides its circuits into those driven primarily by ascending sensory information and those driven primarily by descending cortical signals (Halassa and Sherman, 2019). The former have been called “core” or “first-order” neurons but are herein called “Narrow” because of fairly tight convergence of their inputs and narrow divergence of their cortical outputs, illustrated as vertical projections between an individual sensory (S) cortical area and one thalamic nucleus which receives ascending sensory information at the left of Figure 1 (ellipsis indicates similar organization for all primary sensory cortical areas). The latter have been called “matrix” or “higher-order” neurons but are herein called “Broad” reflecting both their cortical input convergence and output divergence, illustrated as the connections of one thalamic nucleus with multiple sensory, associational (A), and motor (M) cortical areas. It is likely, however, that the Narrow and Broad circuit types reflect a continuum rather than a dichotomy (Wolff and Vann, 2019).

The remainder of this review explores the AI implications of a theory of thalamocortical function put forward by Michael Halassa and colleagues (Rikhye et al., 2018a; Wang and Halassa, 2022), based on the detailed neuroanatomical and electrophysiological studies of S. Murray Sherman and colleagues (Sherman, 2004, 2007, Sherman, 2011, 2016; Theyel et al., 2010; Lam and Sherman, 2011; Sherman and Guillery, 2013). At the risk of over-simplifying, the thalamocortical loops appear to govern how much attention the cortex pays to incoming sensory information (! icon in Figure 1). Such information might: (i) be largely ignored, or (ii) lead to more general activation of the primary cortical area to which it projects (a putative function of the Narrow circuits), or (iii) lead to more widespread activation of other cortical areas, including those responsible for formulating and commanding exploratory actions (a putative function of the Broad circuits). This view is consistent with a computational model of the Narrow circuits involved in transmission of afferent information to cortex, in which the corticothalamic synapses that tend to be located on distal dendrites were shown to strongly complement afferent EPSPs from proximally located synapses (Destexhe, 2000). Elements of Broad thalamocortical function such as the interactions between frontal cortical areas and mediodorsal thalamus are just starting to be incorporated in biologically plausible ANNs to account for humanlike decision-making in which strategies are context-dependent (Rikhye et al., 2018b; Hummos et al., 2022).

Importantly, most thalamic circuits of both types have strong reciprocal connections with basal ganglia (Haber and Calzavara, 2009; Arber and Costa, 2022), a midbrain subsystem that appears to generate value judgments based on experience of rewards and punishments (Schultz, 2016). Thus, decisions to ignore or further analyze incoming sensory signals or to pursue additional sensory signals through active exploration depend on the equivalent of a cost-benefit analysis, consistent with human behavioral strategies in time-constrained perceptual tasks (Smith et al., 1982). In Figure 1 the thresholds for these decisions in the Thalamic Nuclei are modulated by the background activity from the Basal Ganglia. This added functionality accounts for the tendency of human capabilities and failings to be highly dependent on circumstances that are independent of the cognitive tasks themselves such as reward schedules (Fröber and Dreisbach, 2014). The thalamus also receives direct input from the amygdala, particularly to the inhibitory thalamic reticular nucleus (Zikopoulos and Barbas, 2012). Diffuse inhibition from this pathway has been added to the conventional model of thalamocortical transmission of sensory signals to account for cortical attention based on emotional state (John et al., 2016).

The extensive reciprocal connectivity between the thalamus and all cortical areas and the strong dependence of cortical function on signals from the thalamus suggests a theory of computation that leads to more than the sum of the parts. As an analogy, consider the central processing unit of an early computer. It has separate types of circuitry (usually in separate locations) for fetching and storing data and instructions and for registers that interpret the instructions and perform calculations on the data. Combining complete but separate understanding of each type of circuit would not provide useful insights into what the computer can do.

### Attention and saliency

The evolution of the vertebrate central nervous system seems to have been driven by the trade-offs inherent in maximizing the amounts of sensory information available while minimizing the time required to respond effectively to this information flood. If computational resources are limited, then attending to some stimuli implies ignoring others (valve icon in ascending pathway to sensory cortex in Figure 1). If memory capacity is limited, then unattended and evanescent information in the sensory data stream will be irrevocably lost. The sensory features that drive attention are said to be “salient”; careful selection of those features is critical for the survival of the organism.

Salience has been particularly well-studied in the visual system, where it can be divided into bottom-up and top-down features (Li, 2019). Both have been attributed to corticocortical circuits but seem also to involve corticothalamic circuits:

• Bottom-up salience has been attributed to patterns of surround inhibition among feature detectors within the visual cortex. This results in local peaks of neural activity that correspond to regions of the visual field that are heterogeneous (Li, 2022), even if the heterogeneity itself is not consciously perceived (Watanabe et al., 2011). Bottom-up visual flow comes through the lateral geniculate nucleus of the thalamus, which has rich reciprocal connections with the cortical feature detectors. Furthermore, the primary visual cortex has rich reciprocal connectivity with the pulvinar and mediodorsal nucleus of the thalamus, which receive low-level visual information from the superior colliculus. That midbrain structure has its own internal saliency system of reciprocal inhibition that enables it to direct gaze selectively to one of many possible targets (Fecteau and Munoz, 2006).
• Top-down salience denotes the ability of the cortex to use high-level contextual information to increase the responsiveness of lower-level stages to signals that are expected in that context. Again, these can be attributed to long distance, recurrent corticocortical projections but much of that information flows also from prefrontal cortex through mediodorsal thalamus. In the extreme, projections of expectations has the potential to recreate the patterns of neural activity associated with a stimulus that is mentally recalled rather than physically present (Bergmann et al., 2016). Illusory perception occurs normally during dreaming, a function that depends on spontaneous thalamocortical activity.

Salience has been less studied for non-visual senses but it is probably as important. Behaviorally relevant changes of receptive fields to temporospatial patterns of whisker stimulation have been reported in rat somatosensory barrel cortex (Ramirez et al., 2014) and appear to be related to thalamocortical activity (Zhang and Bruno, 2019; Rodgers et al., 2021). Ferrets performing a complex acoustic discrimination task can dynamically retune the spectral receptive fields of neurons in primary auditory (A1) cortex to improve their performance on the task (Fritz et al., 2007). Importantly, expectations based on visual perception can create illusory auditory percepts (Mcgurk and Macdonald, 1976) and expectations based on auditory percepts can create illusory tactile percepts (Jousmäki and Hari, 1998). Such intermodal illusions provided support for a “global workspace” model of conscious attention (Baars, 2007) but they can arise without conscious perception (Ching et al., 2019). During a sleeplike state induced by ketamine/xylazine anesthesia in rats, the intracellular responses of neurons in primary somatosensory (S1) cortex to repetitive patterns of electrotactile stimulation of the paw were modulated by microstimulation of remote cortical areas that generated no direct responses in S1 (Etemadi et al., 2022). The Broad thalamocortical system seems well-suited to such widespread, multimodal integration.

### Active exploration

The common experimental paradigm of stimulus-response biases theories of computation toward a hierarchy of signal processing: temporospatial patterns of primary afferent activity followed by successively more compressed (i.e., abstract) remappings and ending with motor responses. This hierarchy breaks down, however, when corticothalamic circuits are considered from the perspective of continuous behavior involving sequences of exploratory movements and extracted percepts (Loeb and Fishel, 2014). Anterolateral and primary motor cortex and associated ventromedial thalamus have relatively Narrow loops (vertical projections and valve icon at right side of Figure 1). These are anatomically similar to those of primary sensory cortex (Collins and Anastasiades, 2019) but they can be thought of as the highest order of cognitive processing. Thalamocortical activity is necessary for the amplification and release of a voluntary movement plan from motor cortex (Sauerbrei et al., 2020) and for widespread recruitment of sensory cortical areas that might provide feedback to control the movement (Talati et al., 2005). This might arise directly from the Narrow corticothalamocortical loops of motor cortex and/or they might arise in a three component loop from cortex to striatum to thalamus and back to cortex (Şengör et al., 2008). Both would be consistent with the “actor-critic” theory of the role of the basal ganglia in reinforcement learning (Joel et al., 2002). Actor-critic systems that compare new data to model-based predictions have been widely developed for movement sequencing in robotics applications (Ciosek et al., 2019; Clavera et al., 2020).

The decision to engage an exploratory movement will have perhaps the broadest possible effects on perception. These action-perception loops result in a different type of “recurrency” from that discussed below for cortical circuits. The sensory inputs to somatosensory and visual thalamus are strongly dependent on the hand movements and gaze shifts mentioned earlier. The same stimulus features that drive the P300 (rarity and importance) are correlated with widespread orienting reflexes that have both skeletomotor and autonomic components (Donchin, 1981); these constitute physiological indicators of “awareness”. Purely psychological constructs of behavior similarly relate awareness to perception and action (Mackay, 1990).

For historical and methodological reasons, the thalamic nuclei are usually considered in relation to sensory transmission and processing. For historical and clinical reasons, the basal ganglia are usually considered in relation to motor output (as in the akinesia of severe Parkinson’s disease). Both, however, have strikingly similar patterns of connectivity with each other and with all areas of cortex—sensory, associative, and motor. All cortical areas have qualitatively similar, layered circuits both intrinsically and extrinsically (Diamond, 1979).

Motor cortex must interact with the phylogenetically older midbrain tectum, which subserves multimodal sensorimotor exploration and integration in primitive vertebrates that have little thalamocortical function. Even in mammals, the superior colliculus of the tectum is capable of selecting visual, auditory, and tactile targets for attention and initiating coordinated movements to acquire those targets through gaze saccades of eyes and head (Corneil, 2011), ear pinna movements (Stein and Clamann, 1981) (uniquely impoverished in humans), reaching with arms (Pruszynski et al., 2010) and positioning the feet (Weerdesteyn et al., 2004). Orienting reflexes such as gaze saccades and posture shifts originating in either tectum or cortex start out as high-level goals that require kinetic elaboration and coordination by centers in the brainstem reticular formation and spinal cord that are under cerebellar control (Sparks et al., 2001; Loeb, 2021).

The Narrow projections of various anterior thalamic nuclei to frontal cortical areas for limb and eye movement carry collateral discharge activity from superior colliculus and cerebellar deep nuclei (Sommer, 2003; Sommer and Wurtz, 2008). From the perspective of the cortex, this constitutes “sensory” information about the autonomous executive function of these subcortical motor centers. The motor cortical areas might then ignore, attend to, augment or countermand these actions. The thalamocortical system builds upon these subcortical capabilities (bottom half of Figure 1) rather than superseding them. Cortical efferents (not illustrated) project to and modulate all of them (Gallivan et al., 2018; Arber and Costa, 2022; Contemori et al., 2022).

Selecting and performing an exploratory action implies a hypothesis about the likely identity of the unknown entity that is the source of the sensory data. There is a long history of theories of cognition in which the brain formulates and then tests predictions about the entity that might be the source of sensory information, recently reviewed by Siman-Tov et al. (2019). Bayes theorem provides a formal basis for computing the probabilities of various identities of such unknown sources (Bayes and Price, 1763). The thalamocortical system has been proposed as the substrate for the generation of predictions of sensory information based on such probabilities and their comparison with actual sensory signals (Friston et al., 2017a).

Inverting Bayes theorem provides a formal basis for identifying the exploratory action that is most likely to disambiguate the probabilities of those sources (Fishel and Loeb, 2012). The reciprocal connectivity among cortical areas shown by the horizontal arrows in Figure 1 provides a mechanism for aligning the sensory and motor aspects of this hypothesis. The modulatory function of the sensory thalamus provides means to promote those aspects of the ascending sensory information that are most salient for the hypothesis. The sensory cortex must then compare the incoming sensory data to the mind’s eye recall of the salient sensory data to decide if the hypothesis is confirmed or refuted. If these two types of data are insufficiently congruent, a broader consideration of the data is warranted, likely involving more abstract cortical levels (Jordan and Jacobs, 1994). Persistent incongruence requires consideration of alternative sources and, eventually, exploratory actions to acquire additional data. All these processes seem amenable to neuromorphic computing (Friston et al., 2017b) but most have yet to be implemented as such in ANNs.

### Recurrent networks

Most connections between subsystems of the central nervous system are reciprocal rather than unidirectional. Neuromorphic models with such recurrent loop connectivity pose special computational challenges. The recurrent circuits can amplify transients into epileptic-like instability, a problem that can be mitigated by introducing synaptic shunting mechanisms that have a clear basis in neuronal biophysics (Rongala et al., 2021). Learning based on synaptic plasticity in such recurrent, convolutional ANNs tends to result in unphysiological expansion of receptive fields, a problem that can be overcome by adding gates to control spread of excitation (Wang and Hu, 2022).

**Figure 2:**
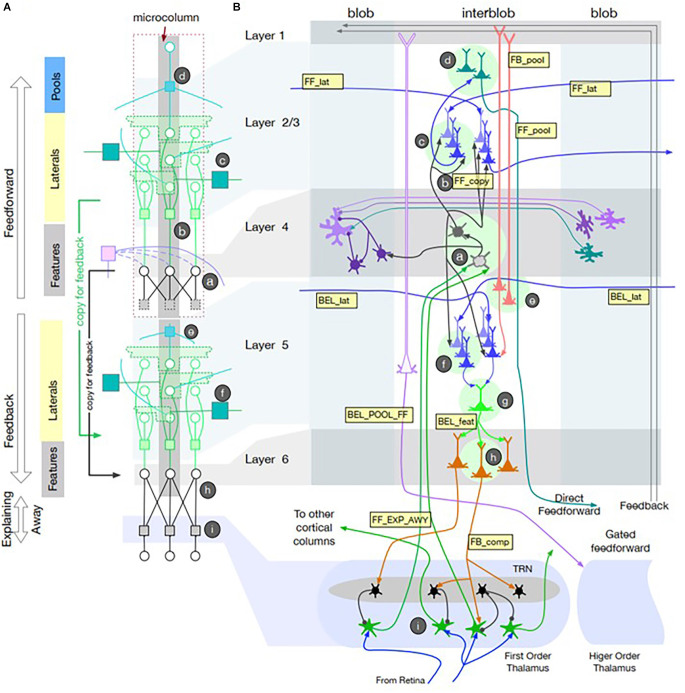
Recursive Cortical Network (RCN) model of primary visual cortex reproduced from George et al. (2020); with permission of the authors. **(A)** Model of microcolumn architecture with separate computational elements for feedforward and feedbackward computations associated with cortical layers 1–4 and 5–6 + thalamus, respectively. It uses factor graph notation for message passing between nodes (circles) via distributed, weighted projections (squares) that are integrated at nodes according to summative or multiplicative functions that can be realized neuromorphically. Messages pass in both directions along lines illustrated according to recursive connections among neurons within the microcolumn. The messages represent belief propagation based on the probability that a given feature is present in the pixel data from the retina. **(B)** Neuroanatomical representation of a model of visual cortex with columns for color detecting “blobs” and contour feature detecting “interblobs.” Lateral connections among Feature detectors allow Pool neurons to generalize contour feature detection across retinal locations. Intracortical feedback circuits generate illusory contours and their filling with adjacent color features. Narrow-type corticothalamocortical feedback results in Explaining Away, whereby partially occluded images are readily perceived because the occluding object provides an explanation for the missing contours. Broad-type corticothalamocortical projections generate Gated feedforward projections to other cortical areas. See George et al. (2020) for mathematical description of the various message passing functions and simulations of these visual perceptual phenomena.

Cerebral cortex contains two different recurrent pathways: corticocortical and corticothalamocortical. Corticocortical pathways such as the well-described surround inhibition among cortical columns appears to account for simple phenomena such as feature detectors (Hubel and Wiesel, 1962) and more complex ones such as inference about partially occluded objects as required by CAPTCHAs (Completely Automated Public Turing test to tell Computers and Humans Apart; George et al., 2017). Adding Narrow-type thalamocortical gates to a detailed neuromorphic model with multiple cortical layers and interconnected columns (see Figure 2) accounts well for some illusory visual percepts that humans generate (George et al., 2020). It provides an anatomically realistic basis for imagined percepts and their comparison with sensory information. The Broad thalamocortical circuits predict that recalled visual images should produce more diffuse excitation of early stage cortical areas than did the original images, which has been reported recently (Favila et al., 2022).

Interestingly, the two main groups publishing theories and models of recurrent thalamocortical function started from different perspectives (Halassa and Sherman, 2019 from academic neuroscience; George et al., 2020 from industrial artificial intelligence) and have not cross-referenced each other. Much of the work in deep-learning AI is structured as factor graphs, a general mathematical tool for factorizing complex functions into local functions operating on limited subsets of variables (Kschischang et al., 2001). Unfortunately, much AI research uses functions such as back-propagation that are not constrained by neuromorphic considerations, although a neuromorphic approximation of back-propagation has been described (Neftci et al., 2017). George et al. (2020) provided neuromorphic realizations of the local functions that they used, which should generate testable hypotheses about neural activity that might be recorded in corticothalamic circuits.

Recurrent or convolutional ANNs have mostly been used to generate fixed sequences such as procedural motor memory (Paine and Tani, 2004). They have been applied to syntactical transformations in language translation, but they can be replaced in that application by training a network to attend to different positions in the input and output syntax (Vaswani et al., 2017). ANNs that employ back-propagation provide an opportunity to use top-down feedback to amplify and effectively attend to those aspects of the input signal that are most salient for a given percept (Zhang et al., 2018). Localized recurrence in reciprocally connected, biologically plausible Hopfield neurons has been suggested to increase the memory capacity of such networks (Krotov and Hopfield, 2021).

Many of the developmental and regulatory details of thalamocortical connectivity probably reflect mechanisms to mitigate instability inherent in recurrent circuits and the consequent clinical dysfunctions to which cerebral cortex appears to be prone. Corticothalamic circuits include more complex synaptic organizations than those found in most ANNs. These include triads with GABAergic inhibitory interneurons (Sherman and Guillery, 2013) and hyperpolarizing metabotropic glutamate receptors (Sherman, 2014). Such details are important for the algorithmic and implementation levels of understanding rather than the theory of computation level considered here, but they provide yet more opportunities for bio-inspired improvement of ANNs, as proposed and demonstrated by George et al. (2020).

## Phenomena to be replicated and explained

### Long-term retention

Artificial ANNs are subject to “catastrophic forgetting” in which the constant adjustment of their gains to recognize new objects tends to degrade old memories of previously experienced objects (Grossberg, 1976; French, 1999). A recent review by a large working group of the inverse challenge of “lifelong learning” identified sets of performance features and mechanisms that appear to account for biological performance and identified examples of ANNs that incorporated analogous mechanisms (Kudithipudi et al., 2022). Many of these involve added executives that replay and refresh old memories (González et al., 2020) or generate new memory elements as needed (Aimone et al., 2009) or enable metaplasticity in only a limited subset of neurons and stabilize the synapses of other neurons after learning (Kirkpatrick et al., 2017; Masse et al., 2018). Simultaneous plasticity in multiple parts of the highly distributed biological nervous system poses an even greater challenge as higher levels contend with changes in the behavior of the lower levels on which they must build (Wolpaw and Kamesar, 2022).

The gating function of the thalamocortical system suggests a strategy to avoid the unwanted plasticity in the first place by blocking the entire ANN from further consideration of input data that are recognized as likely to arise from, hence congruent with, a previously identified entity. The computation of expectations and their comparison with sensory data was first suggested as a solution to this problem in ANNs (Grossberg, 1987) and has been implemented in a modern ANN (Brna et al., 2019). The amount of sensory data that gets to cerebral cortex and the extent of the response to those inputs is modulated by thalamus in response to corticothalamic feedback. If the initially arriving sensory data are sufficiently congruent with current expectations, the thalamus can shut down further processing of the sensory signals, which would greatly limit the effects of any cortical NN plasticity. Thalamic and cortical activity consistent with suppression of context-irrelevant, multimodal sensory cues has been reported (Rikhye et al., 2018b). Conversely, if the initial sensory data are incongruent, the thalamus can sustain and expand the transmission of sensory data to broader regions of cerebral cortex, thereby enabling NN plasticity to integrate multimodal sensory data with the exploratory action that gave rise to these data. The repeated recall of memories of some entities must itself be protected from degrading other, less recalled entities, a requirement that can be met by the separation of the forward and backward message-passing circuits in a model of the thalamocortical system (George et al., 2020).

Central to the theory of computation presented in Figure 1 is the requirement for an incongruency detector. This is a general circuit that is triggered by a failure of the incoming data to be accounted for by the mind’s eye hypotheses about the identity of the source of those data. The P300 is evidence that such a detector exists within the thalamocortical circuitry. One way to embody such a detector in a conventional NN system is to note that the patterns of activity in the output layer of a NN are fundamentally different when the NN is exposed to a novel entity as opposed to one on which it has previously been trained. The latter output pattern will be highly converged on one output element whereas a novel input pattern will generate weak and diffuse activity on many output elements. Uncertainty is correlated with a shift of the EEG from regular alpha rhythms (8–15 Hz) to a more excitable and diffusely active state (Kosciessa et al., 2021). The EEG changes have been interpreted as indicative of thalamocortical activation but they may have started with the different patterns of corticothalamic activity arising from congruent vs. incongruent sensory data.

#### Working hypothesis

The thalamus detects the degree to which ascending sensory information is recognized by cortex as congruent with an existing memory. If the congruence is over a threshold set by the basal ganglia, transmission of the sensory information is blocked, preventing synaptic plasticity from over-writing the existing memories.

### Single-trial learning

The large number of presentations of input data required for ANNs to converge on solutions is usually incompatible with real-world experience, in which it may be impractical or dangerous to continue to experience firsthand the sensory signals associated with a novel and perhaps threatening entity. This might be an argument against the biological validity of the whole “data hungry” ANN enterprise as currently modeled. Another way to address this challenge, however, is to add “snapshot” circuitry (camera icon in middle of Figure 1) that can save the novel input state plus regenerative circuitry to recreate it “in the mind’s eye” (recurrent arrow on camera icon). Such a boost to memory persistence has been attributed to hippocampus (Duszkiewicz et al., 2019), which appears to be a primitive version of neocortex that has similar thalamic connectivity (Aggleton et al., 2010). Thus learning appears to be effected by only one real-time experience with the novel entity, but replaying of the snapshot during conscious recall or dreaming provides the repetition required for synaptic plasticity in the biological NN to recognize and deal appropriately with the entity if encountered again in the future.

Single-trial learning that eventually defines a new category of entities is essentially the extreme opposite of a thalamocortical decision that the sensorimotor data are a good-enough match to a familiar category. If the incongruent sensory data hypothesized above continue to appear to be both novel and important after consideration by the whole of cortex, then rapid long-term potentiation (LTP) in the thalamocortical circuits could effectively record the concurrent activity patterns during the initial presentation. The presence and amplitude of the thalamocortical P300 wave appears to be correlated with the “memorability” of sensory inputs (Donchin, 1981). Various forms of Hebbian post-synaptic plasticity have been identified in cortex for most of the sensory modalities conveyed through thalamus (Heynen and Bear, 2001; Fox, 2002; Shyu and Vogt, 2009; Liu et al., 2011; Audette et al., 2019; Williams and Holtmaat, 2019). The earliest demonstrations of thalamocortical LTP were in motor cortex (Baranyi et al., 1991), which is consistent with the fact that sensory signals cannot be interpreted without considering the exploratory movements with which they are associated (Katz, 1925; Loeb and Fishel, 2014).

The circuits and mechanisms involved in LTP of thalamocortical projections are sufficiently complex and selective to support separation of the general arousal mechanism from the snapshotting mechanism (Audette et al., 2019; Williams and Holtmaat, 2019). The triadic circuits (Sherman and Guillery, 2013) have similarities to the Dense Associative Memory network proposed for Hopfield ANNs (Krotov and Hopfield, 2021).

In addition to recording incongruent sensorimotor data for further consideration, single-trial learning requires a mechanism to replay the recording. Patterned spontaneous activity in adult thalamus constitutes a local persistence of the widespread spontaneous activity found throughout the developing nervous system, which appears to be essential for the initial formation of the anatomically orderly circuits found in adults (Martini et al., 2021). Spontaneous, rhythmic activity in thalamus is conveyed to cortex (Steriade, 1997), particularly during sleep (Steriade et al., 1993). Such activity underlies at least some of the cortical plasticity associated with “memory consolidation” (Chauvette et al., 2012). Rats that are learning to run mazes generate hippocampal activity during sleep that resembles the activity recorded during training while awake (Ji and Wilson, 2007), consistent with the snapshot hypothesis. Ironically, the same mechanism has been proposed to replay and refresh old memories to avoid catastrophic forgetting (González et al., 2020), but that seems to require a constantly growing volume of such replays.

Criteria for snapshotting would usefully include “important” as well as “novel”. The other major input to the various thalamic nuclei comes from the basal ganglia (BG), a midbrain subsystem that receives and learns from reward and punishment experiences. Permissive signals from the BG to the thalamus appear to be necessary for actions and other decisions proposed by all parts of the cerebral cortex to be realized. Damage to the BG is responsible for the well-known pathology of Parkinson’s disease, which includes both difficulties initiating voluntary movements as well as various cognitive disorders related to intention (Solla et al., 2011). Inhibitory output from the BG effectively adjusts the thalamic thresholds for making decisions to ignore, attend to or snapshot the sensory signals, according to the competing costs of erroneous vs. delayed decisions.

#### Working hypothesis

When ascending sensory information cannot be reconciled with any previously learned entity in cortex, the incongruity is detected by thalamus and triggers thalamocortical activity that potentiates currently active cortical synapses. Offline activation of the same thalamocortical circuits then recreates the sensorimotor signals so that synaptic plasticity can eventually learn to recognize and deal with a new entity.

### Illusions

Humans are notoriously bad eyewitnesses. If they choose to attend to one aspect of a scene, they tend to overlook other aspects in plain sight (Simons and Chabris, 1999). They jump to conclusions about what they have witnessed from fragmentary data and they have little sense of any discrepancies between those conclusions and the original sensory data from which they were derived. Those conclusions are highly biased towards expectations derived from circumstantial information that may be irrelevant or misleading (Buckhout, 1974). The top-down saliency mechanism described above includes recurrent pathways from high-level back to low-level cortical representations that are associated with recall, imagination, dreams, and illusions. Such reverse-order processing has been directly demonstrated during recall of music (Ding et al., 2019). As discussed above, long-loop recurrent connections are rarely modeled within ANNs because of instability, but corticothalamic circuits include more complex synaptic organizations that might mitigate such challenges.

One consequence of decision-making based on iterative hypothesis testing is that it requires an uncertain amount of time that depends on the number of iterations. Because there is already a well-formed (but perhaps erroneous) hypothesis to explain the sensory data, it may be better to accept the hypothesis and get on with life. This will be particularly true if the BG have set the thalamic threshold for noticing and reacting to discrepancies fairly high. In fact, the default condition of the BG is a steady inhibition of the thalamus (Goldberg et al., 2013). That is to say, the cortex is enabled to pay attention to the details of the incoming data only when experience has shown that the stakes are high or the incongruencies with prior experience are large. Once the preconceived notion becomes an accepted illusion, any actual sensory data that might refute the illusion are discarded. If the incongruence is above the lack-of-confidence threshold set by the BG, the snapshot function described above can preserve the ascending information that accounts for the incongruence (by contrast, electronic memory is cheap and fast, so an AI machine could easily retain all its raw data, but ANNs generally rely on simple repetition).

The interplay among speed, accuracy, and incongruence can be appreciated in the task of speed-reading or “skimming”—the ability to peruse rapidly and summarize the content of a written document. Many levels of perception, decision-making and active exploration are refined as readers progress from the serial recognition and sounding out of letters as employed by a young child, to the ability to extract the essential contents of an entire book in less than 1 h (Rayner et al., 2016). Readers at all levels use saccades about three times per second to jump to the next bit of text to foveate and decode, but a speed-reader makes large, variable jumps based on internal hypotheses about what the text is likely to say and where that hypotheses might usefully be tested. If a given hypothesis is incongruent with the results of that test, the reader makes a regression saccade back to earlier text that must have been misinterpreted (inability to do so is a fundamental limitation of RSVP technology—rapid-serial-visual-presentation of individual words on a computerized display; Spence, 2002). If the text is somewhat consistent with the reader’s preconceived notions about the topic, the internal representation of the written message will be mostly an illusory pastiche of the reader’s own memories rather than the message intended by the writer. Interestingly, analogous techniques for what is called rapid-reading of the tactile Braille code by blind persons take advantage of the ability independently to move and attend to the various fingers of the two hands to simultaneously acquire and stitch together percepts from multiple locations on the page (Mcbride, 1974). Both of these reading skills depend on learning the syntactical structure of a given language as a set of exploratory actions, a strategy that also underlies the recent successes of natural language processors (Collobert et al., 2011).

#### Working hypothesis

If the ascending sensory information is deemed sufficiently congruent with an expected memory, the thalamic shut-down of further transmission to and processing by cortex results in the illusion that the current experience is identical with the remembered entity.

### Robust generalization

The statement earlier about “robust interactions with unstructured physical environments” needs a closer examination. Compared to an industrial assembly line, a typical home is an unpredictable jumble of objects but they are usually from familiar classes. Dishes with various shapes and patterns might appear by themselves on a countertop, under cups and utensils in a sink or standing on edge in a dishwasher. Towels of various sizes might be folded neatly on racks, hung on hooks or dropped on the floor. Humans have no difficulty identifying an object’s class and understanding what functions the object affords despite such variability of presentation. They can do so equally well with manual exploration in the dark as with gaze saccades in the light. This is a much more robust form of generalization than has been demonstrated in machine vision. It may be the product of representing classes of objects as clustered patterns of associations between exploratory actions and multimodal percepts (Loeb, 2022).

The antithesis of robust generalization is the susceptibility of deep-learning NNs to adversarial attacks, whereby apparently insignificant or nonsensical patterns of sensory input are perceived as consistent with complex memories. By contrast, humans are subject to perceptual errors that are relatively subtle and can be traced to sensible generalization (Coren and Girgus, 2020; George et al., 2020). Humans explore a newly presented object iteratively, with a purposeful sequence of active movements such as gaze saccades and manipulation that tests plausible hypotheses about the probability of its identification. NNs work by “gestalt” perception in which all available data are processed in one pass instead of progressive consideration of various aspects of the high-dimensional data. In their present form, they are incapable of a regressive reexamination of the data in light of incongruous illusions as described above, despite the fact that it is feasible to retain those data indefinitely in electronic memory.

Iterative rather than gestalt perception can be supported by gating mechanisms that focus perceptual attention on different aspects of an incoming data stream and action mechanisms that select and obtain new incoming data streams from a given object. Both appear to be supported by thalamocortical circuits. Such iterative perception provides a means to test hypotheses about spatial relationships among perceptual features of objects to be identified, the absence of which is usually exploited by adversarial attacks (Dujmović et al., 2020; Cheng et al., 2022). Iterative approaches to focusing on small objects in sparse scenes (Bueno et al., 2017) or navigating through cluttered landscapes (Tai and Liu, 2016) have some operational similarities but do not accomplish this goal.

#### Working hypothesis

The Broad recurrent thalamocortical loops cause the cortical representation of a class of objects to consist of all multimodal sensory experiences of the object associated with the exploratory actions that gave rise to the sensory information. After receiving the initial sensory data from a novel object, the thalamocortical loops select a series of exploratory actions, providing a much richer set of sensory data in which to look for similar memories.

### High dimensionality

Current AI systems based on ANN technology suffer from “the curse of dimensionality” (Bellman, 1957). Each of the possible observations of an entity constitutes a dimension in a hyperspace, whether the observations are pixels in an image, sound energy in a spectrum or force on patches of skin. As the dimensionality of the available sensory data about the world increases, ANNs must be trained on exponentially larger datasets. In contrast, human experts become more efficient as they tackle increasingly sophisticated perceptual tasks. The curse of dimensionality is independent of the data compression and dimensionality reduction associated with the process of identifying an instance of an object class. Thalamocortical circuits contribute to such compression (Komura et al., 2013; Schmitt et al., 2017; Mukherjee et al., 2021) without necessarily addressing the training problem. Iterative application of attentional filters for different spatial frequencies of information in ANNs improves the efficiency of processing high-dimensional, multimodal sensory data but still requires massive training sets (Jaegle et al., 2021).

The curse of dimensionality appears to be overcome by iterative hypothesis testing and decision making (Fishel and Loeb, 2012; Loeb, 2022), which may be an emergent property of the recurrent thalamocortical circuitry. Bayesian inference uses the data from one of many possible observations to adjust the probabilities of various possible identities of the unknown entity. This requires a stored database that provides information about the probability of obtaining a given data value from each of those entities, but it does not require experience with all possible combinations of observations. If inference from that single datum does not shift the probabilities to a definitive identification (i.e., the expectations are incongruent with the sensorimotor data), the inverse process of Bayesian exploration uses the same stored database to identify which next observation to make. The data from that observation is then used in an iteration of Bayesian inference. These alternating, inverse processes continue until the probability of one causative entity surpasses some threshold, whose level should reflect the relative costs of error vs. delay. Importantly, the order in which the successive observations are performed is not fixed; it depends instead on the values returned by the observations that drive the evolving probabilities. Regressive repeats of previous observations are useful and have been observed when identifying entities that are quite similar to each other (Fishel and Loeb, 2012).

The internal representation of any entity can be described as the centroid of clusters of previous observations in the hyperspace of all sensory dimensions and the exploratory actions that gave rise to them (Loeb and Fishel, 2014). This can be implemented as a NN whose input layer consists of various observations and whose output layer identifies entities that are mapped to these clusters. The number of possible observations is very large, but this can be addressed by multilayering, a form of vertical integration. This is now common in deep-learning ANNs (Hinton, 2012) and has a biological counterpart in hierarchical abstraction via multiple cortical areas that successively process and integrate data from multimodal sensors (Fuster, 2006). It is also important to integrate data horizontally across dimensions related to various sensory modalities and exploratory movements. The “Multinet” NN architecture provides this function across visual tasks by adding recurrent circuits that are consistent with corticothalamocortical loops (Bilen and Vedaldi, 2016).

If repeated observations of a novel entity are incongruent with all current output neurons, this new observational cluster will eventually drive Hebbian plasticity to map to a new output neuron, perhaps by subdividing a population of output neurons that was previously tuned to the nearest cluster. Decision-making to adapt an existing memory or create a new one has been observed and modeled for human subjects dealing with perturbations to previously learned sensorimotor tasks (Oh and Schweighofer, 2019). The repeated observations required for either process might arise from replaying a snapshotted set of inputs rather than real-time data from the novel entity, as noted above. This affords opportunities to mix the data from one novel entity with those of others in combinations that are not physically realizable but which may facilitate the detection of common patterns (Seligman, 1987).

#### Working hypothesis

Thalamocortical circuitry recruits the motor and multimodal sensory cortical areas that together contribute to the sensorimotor associations that are the memory of all the experiences with that entity. New experiences that are not sufficiently congruent with the collectively sparse representation of all possible combinations of this multimodal information are repeated until a new associative memory is formed. The thalamocortical circuits cause the cortex to ignore the sensory dimensions that are congruent with previous experience and focus on those that are novel and distinctive.

## A unifying framework

The murky relationships among reality, memory, and illusion have been the subject of much philosophical thought throughout the history of various cultures^1^. Much of the discussion has been informed by introspection, resulting in a circularity of logic that has further troubled philosophers and psychologists (Schwitzgebel, 2019). As engineered systems for AI aim to replicate human cognition, they will start to exhibit behaviors that have similarities to what humans call awareness and consciousness. These will be the emergent properties of circuits that are fully known and testable, finally providing an escape from introspection. At what point will we be comfortable applying these subjective and introspective descriptions of human behavior to such engineered systems or, conversely, accepting that these machines embody human consciousness?

When confronted with a system of the complexity of the CNS, it is natural to break it into manageable subsystems. The distinctive anatomy and histology of different parts of the brain provided an obvious starting point. The distinctive pathology produced by selective lesions of such parts provided hints about function. The concurrent rise of machines that could perform some of those functions led to trying to find correspondences between the anatomical parts of the brain and the block diagrams of engineered systems, a common starting point for theories of computation as defined by David Marr (1982). The methodology of experimental neuroscience and the rapidly growing scale of the literature, however, pushes individual neuroscientists to focus on individual subsystems of the CNS and the neural activity that is found there that correlates with experimental observations of very specific behaviors. It is then natural to apply that local knowledge to build bio-inspired machines that can reproduce those observations. This was the original and ongoing inspiration for ANNs that could perform perceptual tasks similar to those of cerebral cortex. It has been the basis for theories of the spinal cord based on oscillators and servocontrol, of the cerebellum based on error correction, of the basal ganglia based on value and cost functions, etc.

A theory of general intelligence need not have functional boxes that are isorepresentational with anatomically distinct areas of the CNS. The interconnections among such areas are much more dense and recurrent than those of engineered systems, suggesting that much functionality arises from their interactions (Goertzel, 2014). As discussed here, bio-inspired, AI system designs that include recently proposed functions of thalamocortical circuits may account for many human capabilities and limitations that have been difficult to reproduce in ANNs based solely on models of cerebral cortex. The close integration of thalamus and cortex, two CNS subsystems with very different internal architectures, seems to give rise to computational behaviors that are more than the simple sum of the functions inferred from each in isolation. Further integration with other subsystems such as basal ganglia and tectum seems likely to result in similarly nonintuitive emergence of systems behaviors. One barrier to such integration is the fragmentation of research into the disciplines and subdisciplines of neuroscience and those of AI (much of which now occurs in commercial entities), that have largely independent conferences and journals.

The emergent behaviors of complete neural systems include quirks that arose as evolutionarily useful accidents whose benefits once exceeded their costs. Later evolving parts of the CNS then had to contend with these quirks. Such phylogenetic development likely results in overall performance and individual mechanisms that an engineer designing a system from scratch would eschew (Partridge, 1982). If the goal is to discover the basis for human intelligence rather than to invent a better machine, then a model system should have subsystems, connectivity and emergent capabilities and limitations analogous to those of humans. From that perspective, it does not matter whether the phenomena discussed above are viewed as features or bugs. They must arise from the design and function of a physical neuronal system, so they can and must be produced by candidate computational models of this system.

To be sure, the history of theories of neural control based on analogy to engineered technology contains many disappointments (Loeb, 2021), but that, too, is progress. The hypothesis testing of the scientific method is not fundamentally different from that of everyday life:

“When you have eliminated the impossible, whatever remains, however improbable, must be the truth.” Sir Arthur Conan Doyle, stated by Sherlock Holmes.

## Author contributions

GL is solely responsible for this article.

## Footnotes

^1^ https://en.wikipedia.org/wiki/Reality
